# Maternal Exposure to Mycoplasma Pneumonia and Amniotic Constriction Band: A Case Report of Probable Novel Etiology

**DOI:** 10.7759/cureus.31410

**Published:** 2022-11-12

**Authors:** Ahmed Aljabali, Asmaa Eltobgy, Sarya Swed, Own Khraisat

**Affiliations:** 1 Faculty of Medicine, Jordan University of Science and Technology, Irbid, JOR; 2 Department of Obstetrics and Gynecology, Al-Azhar University, Cairo, EGY; 3 Faculty of Medicine, Aleppo University, Aleppo, SYR; 4 Faculty of Medicine, Al-Balqa' Applied University, Salt, JOR

**Keywords:** infection, mycoplasma pneumonia, auto-amputation, amniotic constriction band, case report

## Abstract

Amniotic constriction band (ACB) is an uncommon clinical concept with different presentations specific to each patient with clinical symptoms may include ring constrictions, digital defects, natural limb amputations, and visceral defects. The etiology of this defect is not fully understood.

We present a full-term newborn boy who was born by vaginal delivery to a healthy mother. At birth, amniotic bands encircled and constricted his upper and lower limbs. At two and six months of gestation, the mother gave a unique obstetric history of recurrent exposure to her infected daughter, which was diagnosed later as a case of atypical *M. pneumoniae*. This raises suspicion that *M. pnemoniae* may play a critical role in the pathogenesis of ACB and the hypothesis related to its origin.

The inquiry in our case is whether *M. pneumoniae* might have been a non-aberrant teratogen and caused subclinical chorioamnionitis that leads to early rupture of amniotic membranes and result in the proposed defects. As far as we know, this is the first case reported in the literature that combines gestational exposure to *M. pneumoniae* and postpartum isolated amniotic constrictions and minor digital defects in Saudi Arabia newborns. In addition, we discussed the possible underlying causes and reviewed the published literature on this defect.

## Introduction

Amniotic constriction band (ACB) is a group of congenital disorders marked by a wide range of clinical presentations, from simple ring constrictions to multiple congenital abnormalities incompatible with life [1]. In live birth, on average, occurs every 1,200 to 15,000 newborns, whereas spontaneous abortions occur every 178 to 10,000 fetuses [2]. Most cases are sporadic and affect both genders equally [3]. African Americans are reported to suffer from these defects 1.76 times more frequently than Caucasians [4].

Despite extensive research, ACB's exact etiology has not been determined. Considering, the variation in clinical characteristics among patients with the unusual nature of this constellation of findings, besides unknown ACB's exact etiology, it is fitting that exact synonyms to this entity varied and are described in 34 synonyms, including “aberrant tissue band syndrome, amnion rupture sequence, constriction band syndrome, and Streeter's dysplasia.” The term “syndrome” is debatable because there are no characteristic, persistent, and distinguishing signs of the ACB, as in other syndromes. Based on literature reviews, it was determined that ACB was the best term to describe the etiology and manifestation of this disease [5]; accordingly, we adopted the same term here.

ACB is generally associated with deformations, malformations, and anomalous disruptions. Disrupting effects can occur when normal tissue breaks down, including constriction bands, amputations, and acrosyndactyly. During gestation, an insult can cause malformations or abnormal organ development. Various abnormalities and multiple extremities are typical phenotypes of the ABC.

The most common abnormalities include constriction rings, amputations, and acrosyndactyly [6]. It has been debated why the ACB develops, but no robust findings have been reached. Each theory has limitations (intrinsic, extrinsic, vascular, and others). As the discussion proceeds, some authors have adopted the notion that no single explanation can encompass all manifestations [7]. Although the catastrophic effects of microbial infections on the developing baby, little is documented about the mechanism by which pathogens overcome the placental barrier during gestation causing congenital illness [8]. *M. pneumoniae* inhabits the upper respiratory tract and is predominantly responsible for pneumonia in children and adolescents [9,10], causing 10%-40% of pediatric respiratory infections [11]. The infection can infiltrate the host's immune system via different pathways; affected individuals frequently neglect symptoms that lead to missed healthcare diagnoses [12]. pulmonary *M. pneumoniae* is accompanying a typical rash and mucosal infection [13]. Less is known concerning mycoplasma vertical transmission, even though congenital disorders accompany mycoplasma in placenta specimens in newborns [14].

This case involves a preliminary diagnosis of* M. pneumoniae* transmission in the first and second trimesters, followed by the birth of a neonate with ACB characteristics. Despite the absence of isolation of Mycoplasma species from the newborn and mother during gestation, the mother had experienced non-specific clinical signs and symptoms of acute febrile illness during her first and second trimesters only after being exposed to her diagnosed infected daughter with *M. pnemoniae*. The daughter's serological antibody screening test was positive. As far as we know, this is the first case reported in the literature that combines gestational exposure to *M. pneumonia *and postpartum isolated amniotic constrictions and minor digital defects in Caucasian newborns.

## Case presentation

**Figure 1:**
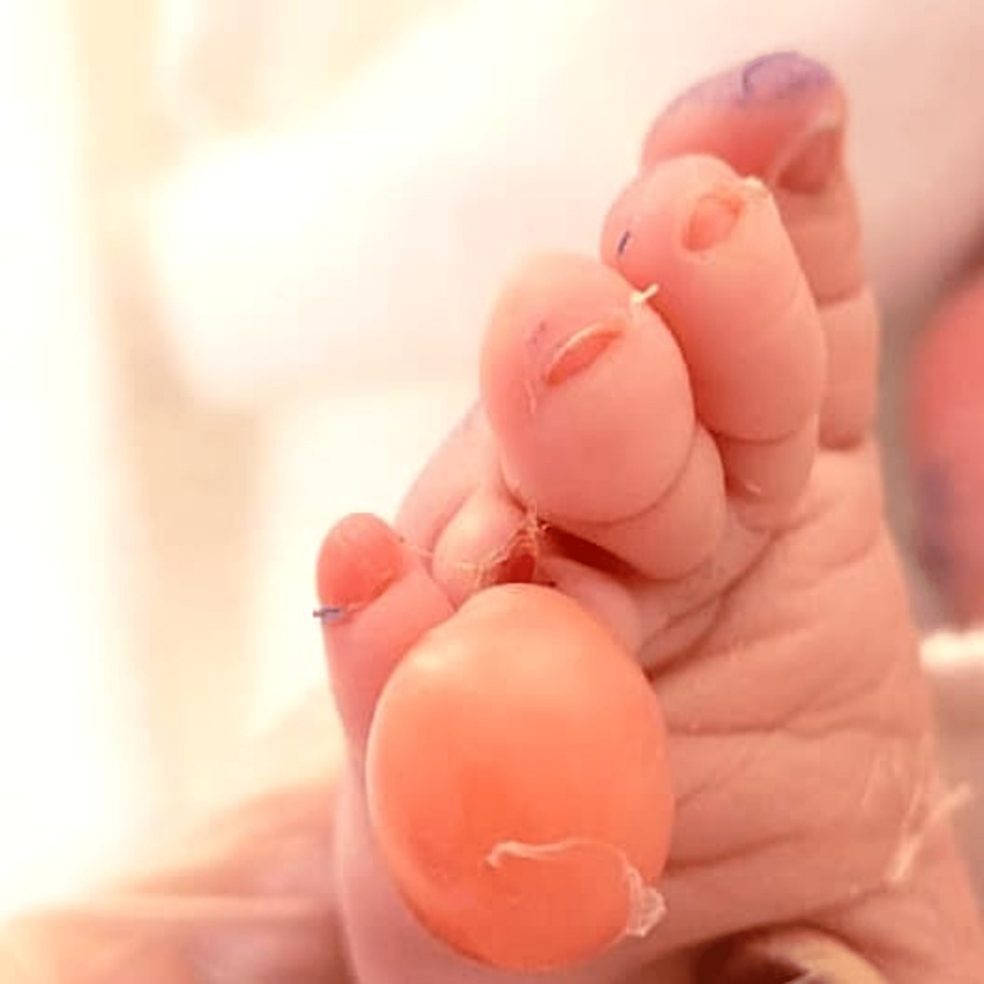
Dorsal view of the left foot shows marked constriction ring at the fourth toe with edema and bullae distally.

**Figure 2:**
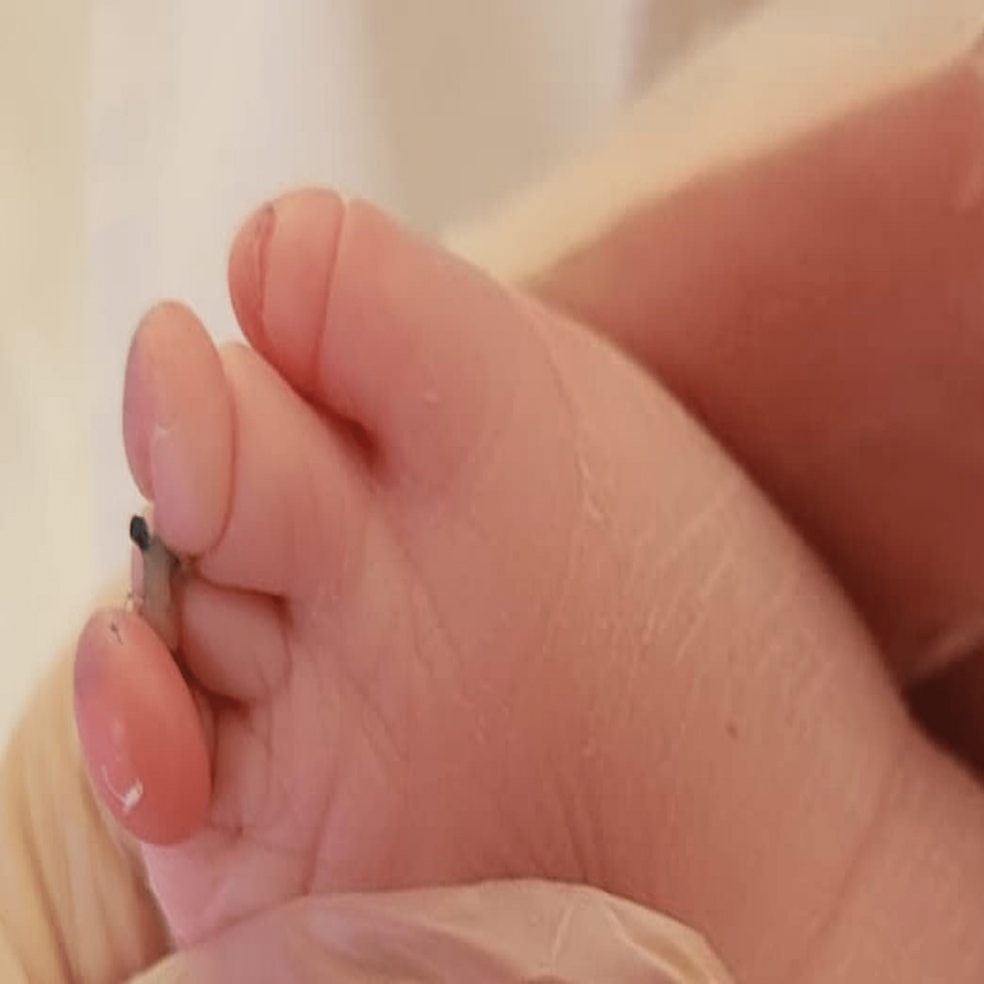
Dorsal view of the right foot shows complete aplasia of the big toe, and the absence of distal phalanx of the third toe and a marked constriction at second and fourth toe with distal edema and deformity.

**Figure 3:**
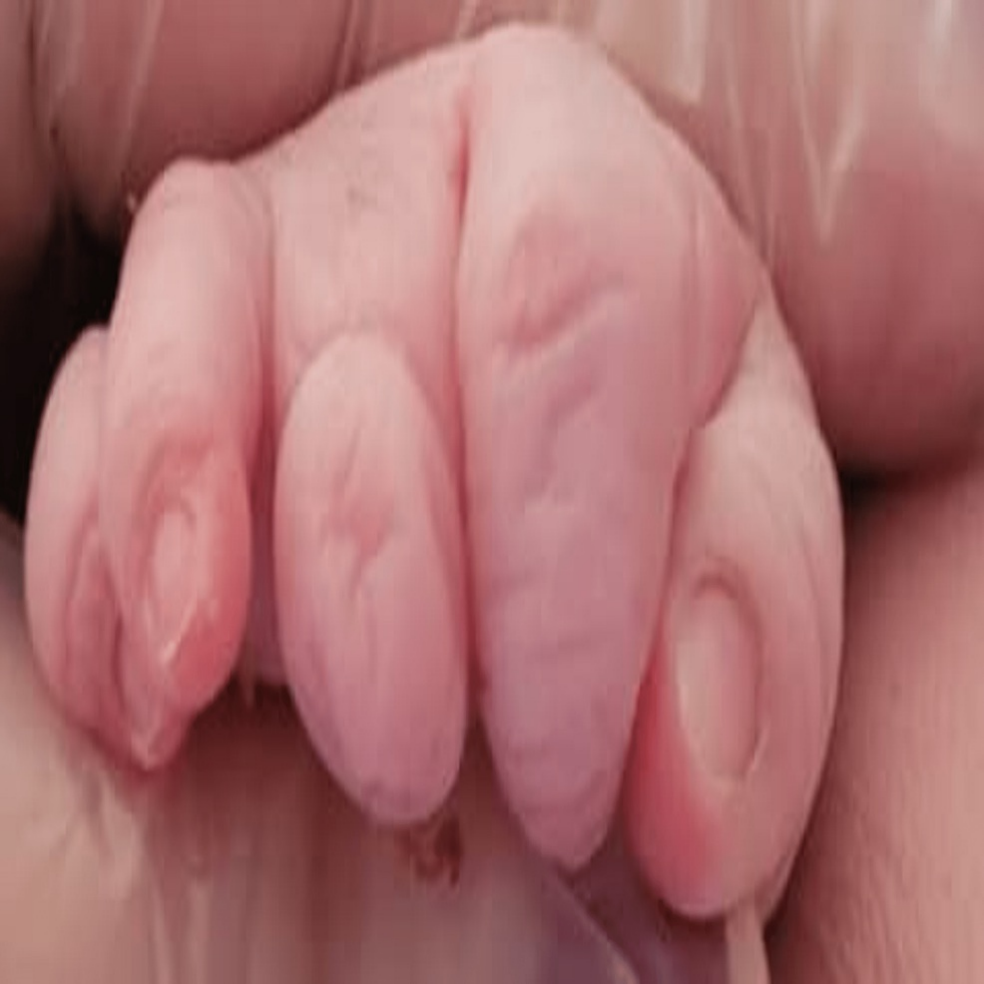
Dorsal view of the right hand shows the absence of the distal phalanx of the long finger.

**Figure 4:**
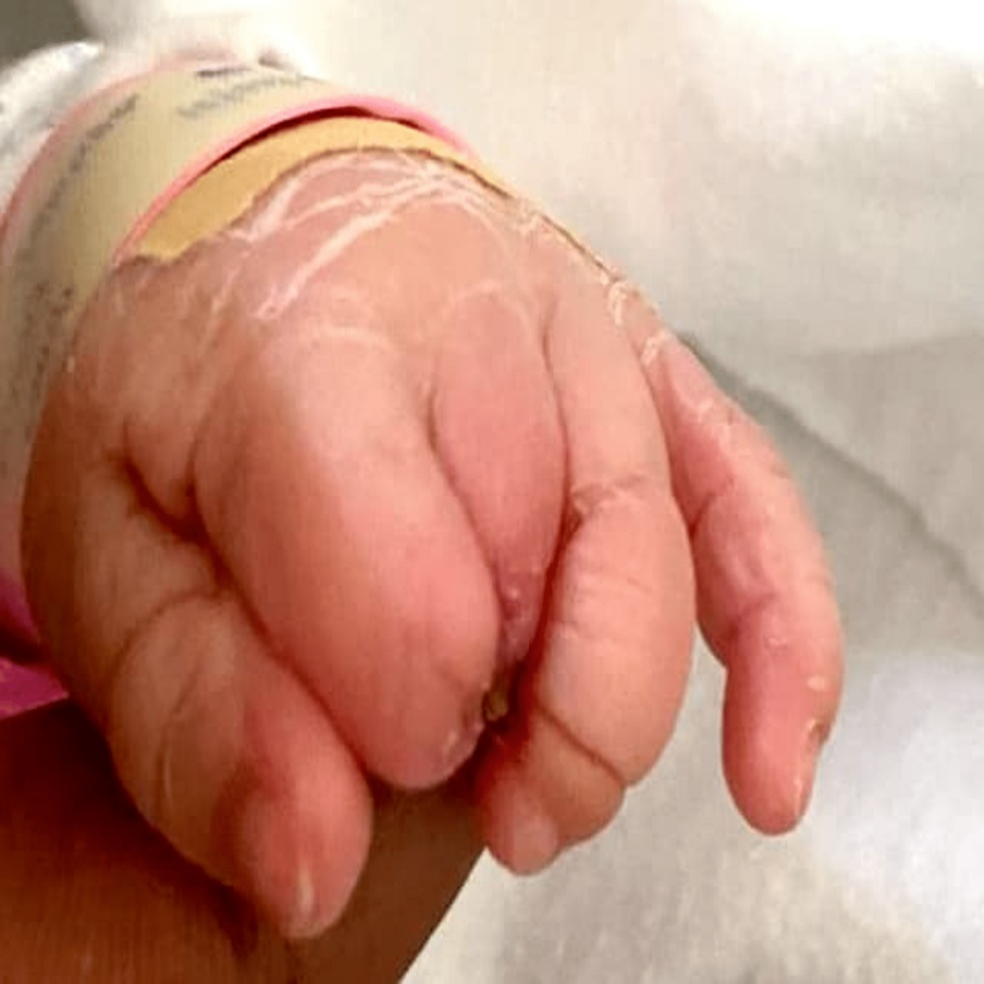
Dorsal view of left hand shows the absence of distal phalanxes of the index, long fingers and superficial band in the ring finger.

A 36-year-old pregnant female, G6P5+0, presented to our unit in labor. She delivered a full-term boy weighing 3.3 kg by vaginal delivery at 39-4/7 weeks of gestation with multiple defects in fingers and toes (Figures 1-4).

He was the fifth child born to non-consanguineous Saudi-Arabia parents after a normal pregnancy. The perinatal period had been uncomplicated, except for routine iron supplements to avoid anemia and lack of any premature watery leakage, trauma, or adverse events throughout her pregnancy. Her past obstetric history was smooth for her previous pregnancies. Without any other congenital defects in the family pedigree, the other four siblings, one male, and three females, were normal. At six weeks of gestation, the mother exposed to her third child, a girl aged 12 years old, who started to develop nonspecific manifestations of upper respiratory infection (URT) in the form of sore throat, bone aches, low-grade fever, multiple painful oral blistering bulla, and ulcers. The mother sought medical advice, and the initial investigation was done (Table 1). The girl was misdiagnosed and mistreated as having an oral herpes infection.

**Table 1:** Girl investigation

Reference Range	Result	Test (initial)
4 -11×109/L	6.44×109/L	WBC
	Negative	Throat swab culture
0-200 IU/ml	238 IU/mL	ASO
	Negative	sickling cell test
0-10 mm/hr	14 mm/hr	ESR (first hour)
Reference Range	Result	Test (after six month)
negative≤0.90, equivocal=0.91-1.09, positive≥1.10	positive	Serology Abs of M. pnemoniae
	2.83 U/mL	IgG
negative˂770 U/ml, low positive=770-950 U/ml, positive˃950 U/mL	1840 U/mL	IgM

**Figure 5:**
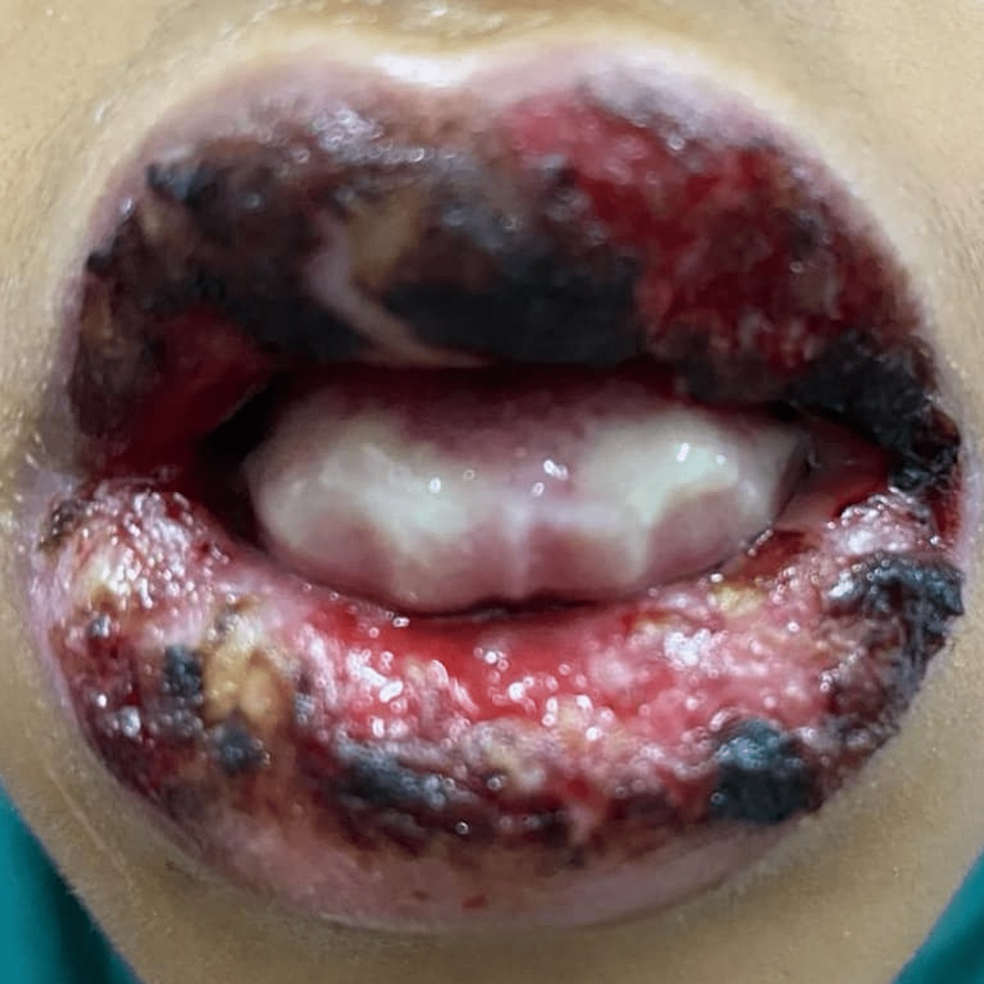
M. pneumonia infection with multiple stages at different times ranging from multiple ulcers, erosion, and hemorrhagic crusts on the lower and upper lip with blistering inside the inner mouth.

After two weeks, the condition resolved spontaneously. During this period mother developed nonspecific manifestations similar to the common cold and resolved after a few days. Her investigation showed in Table 2. After about six months, the child suffered from the same clinical signs and symptoms, especially the same pattern of multiple oral ulcers and blisters, as shown in Figure 5. The mother was 33 weeks' gestation and the only one dealing with her sick daughter; at this time, the physician asked for an antibodies screening test for *M. pneumoniae,* which was positive (Table 1).

**Table 2:** Mother investigation

Reference Range	Result	Test
12 mm/hr	45 mm/hr	ESR (first hour)
6.24-137 ng/mL	177 ng/mL	S. ferritin
1-6 mg/L	5 mg/L	C-reactive protein (CRP)

After delivery, the placenta showed no abnormalities, and the newborn exhibited isolated features of ACB in both fingers and toes. Multiple investigations were carried out to exclude any other congenital anomalies. No other congenital defects were isolated. Informed consent was obtained from the patient's parents.

## Discussion

ACB is a rare congenital illness that affects both sexes equally, has an unknown family association, and manifests as either complex multisystem abnormalities or minor flaws that might go unnoticed during a prenatal ultrasound scan and are only discovered after the baby is delivered [15]. Although amniotic band syndrome has been identified for more than 300 years, there is still no sufficient pathogenic explanation. However, the synthesis of fibrous amniotic bands is often recognized in the obstetric literature as the initial event that causes the condition. It is consequently related to the subsequent pathologic events (exogenous theory) [16]. This hypothesis states that ACB arises from temporary oligohydramnios that results from amnion rupture. Amputations, constriction rings, and skin abrasion can occur as a result of embryonic body parts being entangled with the comparatively “sticky” mesoderm on the chorionic surface of the amnion during the embryo's passage through the amniotic defect [17], the extraordinarily variable phenotype in ACB has been related to differences in the timing of the amniotic rupture [16]. This exogenous hypothesis has primarily replaced the intrinsic defect hypothesis of germ plasma advocated by Streeter [18].

Direct observation of the nature of the placental membranes and fetal abnormalities has made most authors support a mechanical cause [19]. The sporadic incidence of ACB and the absence of accompanying internal anomalies have also been utilized to support the extrinsic theory [20]; this is in line with our case. Since there is no known cause for the original rupture, the creation of bands and other “non-disruption” anomalies suggest the possibility of subclinical infection of *M. pneumoniae*, which is supported by this newborn case and some further findings in the literature. In this instance, amnion rupture was caused by pneumoniae exposure during pregnancy, which may have been the major cause. The genitourinary tract may be infected intrauterine during pregnancy by ascending routes. This resulted in the bacteria surviving in amniotic fluid (AF) and ultimately being transferred to the fetal lungs. In the first trimester of pregnancy, while the fetal membranes remain intact, the mother might get an infection that spreads along the pregnancy [21]. Clinical intrauterine infection and/or the histological manifestation of chorioamnionitis are referred to as chorioamnionitis [22]. the chorioamnionitis in our instance resulted from a previously unknown ascending infection or from a respiratory tract infection that was transferred to the placenta by circulation. The defensive mechanisms of the placenta that prevent pathogens from reaching the fetus, however, remain primarily unknown [8]. By the latter, *M. pneumoniae *can travel via the bloodstream during or after a respiratory tract infection and may result in extrapulmonary symptoms [23]. Mycoplasmas are known to colonize mucous surfaces as mucosal pathogens [24]. Primarily *M. pneumoniae* is well known to invade the respiratory system [25]. *M. pneumoniae* should be considered when making a diagnosis of community-acquired pneumoniae [26]. It may take one to three weeks to develop and spreads via aerosols when individuals cough. *M. pneumoniae* requires close and persistent contact to adjust temperature and humidity [27]. Researchers have discovered that pneumoniae often has a benign course and affects 3- 10% of infected people [28]. Although the respiratory system is the primary location of infection with *M. pneumoniae*, any organ may be affected. Acute extrapulmonary complications may occur in around 25% of hospitalized *M. pneumoniae* patients at some point throughout the illness. These consequences may develop before, during, or after pulmonary signs or even without any respiratory symptoms; the pathophysiology of these issues is unclear [29]. The clinical course of *M. pneumoniae* infection is often regarded as mild, self-limiting, and straightforward. Clinical advancement happens gradually, then radiological advancement. The resistance to *M. pneumoniae* is short-lived, and recurrence is frequent [28]. Identifying the etiology of childhood pneumonia is one of the most significant challenges. The first treatment of choice in daily practice is empirical. In 20%-60% of cases, the etiological agent cannot be identified [30]. Culture, antigen detection, serological testing, and nucleic acid amplification are standard procedures for detecting *M. pneumoniae* [31]. It is apparent that IgM-type antibodies rise after the first *M. pneumoniae* infection, but their increase is low in patients re-infected with the pathogen [32].

## Conclusions

Following the detection of an ACB, an obstetrician should conduct a comprehensive clinical examination to search for any further abnormalities and obtain a full pregnancy history that includes any possible teratogenic exposures and a multigenerational pedigree. Depending on the type of ACB, treatment options may include genetic counseling and surgical referral to improve limb function. To prevent a natural amputation in the case of an isolated amniotic band with a restricted limb, in utero, lysis of the band may be an option. As early as prenatal diagnosis, such therapy is not widely available in Middle Eastern countries.
